# An Overview of Systematic Reviews of *Ginkgo biloba* Extracts for Mild Cognitive Impairment and Dementia

**DOI:** 10.3389/fnagi.2016.00276

**Published:** 2016-12-06

**Authors:** Hong-Feng Zhang, Li-Bo Huang, Yan-Biao Zhong, Qi-Hui Zhou, Hui-Lin Wang, Guo-Qing Zheng, Yan Lin

**Affiliations:** ^1^Department of Neurology, The Second Affiliated Hospital and Yuying Children's Hospital of Wenzhou Medical UniversityWenzhou, China; ^2^Department of Rehabilitation, The Second Affiliated Hospital and Yuying Children's Hospital of Wenzhou Medical UniversityWenzhou, China

**Keywords:** *Ginkgo biloba* extracts, dementia, Alzheimer's disease, vascular dementia, mild cognitive impairment

## Abstract

*Ginkgo biloba* extracts (GBEs) have been recommended to improve cognitive function and to prevent cognitive decline, but earlier evidence was inconclusive. Here, we evaluated all systematic reviews of GBEs for prevention of cognitive decline, and intervention of mild cognitive impairment (MCI) and dementia. Six databases from their inception to September 2015 were searched. Ten systematic reviews were identified, including reviews about Alzheimer's disease (*n* = 3), about vascular dementia (*n* = 1), about both Alzheimer's disease and vascular dementia (*n* = 2), about Alzheimer's disease, vascular dementia and mixed dementia (*n* = 3), and a review about MCI (*n* = 1). Based on the overview quality assessment questionnaire, eight studies were scored with at least 5 points, while the other two scored 4 points and 3 points, respectively. Medication with GBEs showed improvement in cognition, neuropsychiatric symptoms, and daily activities, and the effect was dose-dependent. Efficacy was convincingly demonstrated only when high daily dose (240 mg) was applied. Compared with placebo, overall adverse events and serious adverse events were at the same level as placebo, with less adverse events in favor of GBE in the subgroup of Alzheimer's disease patients, and fewer incidences in vertigo, tinnitus, angina pectoris, and headache. In conclusion, there is clear evidence to support the efficacy of GBEs for MCI and dementia, whereas the question on efficacy to prevent cognitive decline is still open. In addition, GBEs seem to be generally safe.

## Introduction

Cognitive decline is a major social problem of public health (Hugo and Ganguli, 2014; Hill et al., 2015). Cognitive decline includes mild cognitive impairment (MCI) and dementia (Howieson, 2016; Jørgensen et al., 2016; Mormino and Papp, 2016; Ströhle and Rapp, 2016; Thomas, 2016). MCI is characterized by a slight but noticeable decline in cognitive function, which is regarded as a symptomatic stage before progression to dementia (Budson and Solomon, 2012; Thung et al., 2015; Fernández-Blázquez et al., 2016; Petersen, 2016). MCI is further categorized into MCI due to Alzheimer's disease (AD) and MCI due to other causes. Dementia is a chronic acquired progressive mental retardation syndrome (Damiani et al., 2014; Noel-Storr et al., 2014; Ihl et al., 2015; Mitchell, 2015; Ngo and Holroyd-Leduc, 2015; Wang et al., 2016). The causes include AD, vascular dementia (VD), and mixed dementia (Montine et al., 2014; Altamura et al., 2016). AD is the most common neurodegenerative disease, and generally begins with mild memory problems, progressing to the development of multiple cognitive and functional impairment within a few years (Brooker et al., 2014; Dubois et al., 2014; Aygün and Güngör, 2015; Apostolova, 2016; Wood, 2016). VD is a severe cognitive dysfunction syndrome caused by ischemic stroke, hemorrhagic stroke, and cerebral vascular disease with low cerebral perfusion which leads to the impairment of memory, cognition, and behavior (Tsivgoulis et al., 2014; O'Brien and Thomas, 2015). Mixed dementia means that both AD and VD occur in one patient (Moore et al., 2014; Bogolepova, 2015; Kim et al., 2016). Currently, more than 46 million people live with dementia worldwide, and the number is estimated to increase to 131.5 million by 2050 (Prince et al., 2015). Dementia also has a huge economic impact. The total worldwide cost was US$ 818 billion in 2015, US$ 604 billion in 2010, and is estimated to be 1 trillion in 2018 (Prince et al., 2015).

The treatment for dementia and MCI is still symptomatic (Fitzpatrick-Lewis et al., 2015). Up to now, no disease-modifying therapy has been available (Kennedy and Sud, 2014). Cholinesterase inhibitors increase the concentration of neurotransmitter in the brain and improve memory (Rockwood et al., 2013; Yáñez and Viña, 2013; Chen et al., 2016); NMDA receptor antagonists reduce neurotoxicity through inhibiting excitatory amino acid receptors (Newport et al., 2015; Schmidt et al., 2015); other drugs such as Serotonergic agents, Dopamine blocking agents, Benzodiazepines alleviate specific other symptoms (Schneider et al., 2014; Deardorff et al., 2015). Thus, there are multiple alternative strategies with moderate efficacy for some patients for a limited amount of time.

*Ginkgo biloba* extracts (GBEs) are widely used for various kinds of disorders, including cognitive dysfunctions, headache, tinnitus, vertigo, inattention, mood disturbances, cardiovascular diseases, and coronary heart disease (DeFeudis and Drieu, 2000). *Ginkgo biloba* contains flavonoids, terpene lactones, and ginkgolic acids (Oken et al., 1998). GBEs have been demonstrated to have antioxidative activities, to increase tolerance to hypoxia, and to improve blood rheology by increasing the flexibility of cellular blood components, thus enhancing microcirculation; affecting neurotransmitter levels; enhancing neuroplasticity; prevention of brain edema; and neuroprotection (DeFeudis and Drieu, 2000; Tchantchou et al., 2007, 2009; Fehske et al., 2009; Yoshitake et al., 2010; Altamura et al., 2016). GBEs have been widely used in the treatment of dementia for decades now (Weitbrecht and Jansen, 1986; Oken et al., 1998). Some systematic reviews have been conducted to assess GBEs in prevention and treatment of MCI and dementia (Janssen et al., 2010; Wang et al., 2010; Weinmann et al., 2010; Yang et al., 2011, 2014, 2016; Hu et al., 2013; Jiang et al., 2013; Gauthier and Schlaefke, 2014; Tan et al., 2015). To provide an overview over the large amount of available data, we here provide a systematic review of those systematic reviews.

## Methods

### Search strategy

Systematic reviews and meta-analyses of GBEs for prevention, MCI, and dementia were searched in six databases from their inception to September 2015. The databases included Chinese biological medical literature database, Web of science, Pubmed, Chinese Wanfang data, Chinese VIP information, and Chinese national knowledge infrastructure. The search terms used were [“Ginko biloba” OR (“*Ginkgo biloba*” AND extract) OR “EGb 761”] AND (“cognitive function decline” OR “mild cognitive impairment” OR “mixed dementia” OR “Alzheimer's disease” OR “vascular dementia”) AND (“systematic review” OR “meta-analysis”) in English and Chinese. The reference lists of all relevant articles were searched for additional studies.

### Inclusion criteria and exclusion criteria

Studies that met all of the following criteria were included: (i) the articles were systematic reviews and/or meta-analysis; (ii) the publications reported the use of GBEs for prevention of cognitive function decline, or for intervention of MCI, and dementia, including AD, VD, or mixed dementia; (iii) studies were limited to humans.

Studies were excluded if they were any of the following: (i) systematic reviews evaluating other Chinese herbal medicine or mixed treatments; (ii) non-systematic reviews, comments, and overviews.

### Study selection and data collection

All articles were read by two independent reviewers (YBZ, HFZ) and data from the articles were extracted and validated according to predefined criteria. The following details of the article were extracted from the included studies: (1) first author's name and the publication year; (2) the types of diseases, number of primary studies, and quality of primary studies; (3) the conclusions and meta analyses of primary studies, and the nature of the extracts of *Ginkgo biloba*; (4) the overall scores and each items on OQAQ; (5) quality assessment, quality of randomized controlled trials, inclusion criteria and exclusion criteria of primary studies. Data for meta-analyses were based on the reported summary statistics for each study. Disagreements regarding inclusion and quality were settled through discussion or consultation with the corresponding author.

### Quality assessment

Two investigators independently (YBZ and HFZ) assessed the methodological quality of all included systematic reviews by using the overview quality assessment questionnaire (OQAQ) (Al Faleh and Al-Omran, 2009). The OQAQ is composed of 10 questions, graded on a 7-point scale. Questions 1–9, which were answered with “adequate,” “inadequate,” or “not mentioned,” addressed the five methodological aspects of systematic reviews, including search strategy, study selection, validity assessment, data analysis, and conclusions. Only the answer “adequate” receives a positive score, while “inadequate” or “not mentioned” scored 0 point. Each of questions 1–4 and 9 values 1 point, while each of questions 5–8 is 0.5 points. The final question 10 is to conclude the whole points of the scientific quality of these reviews we rate. A score of three or less was regarded as indicative of extensive or major flaws and a score of 5 or more as suggesting minor or minimal flaws.

## Results

### Description of the screening process

**Figure 1:**
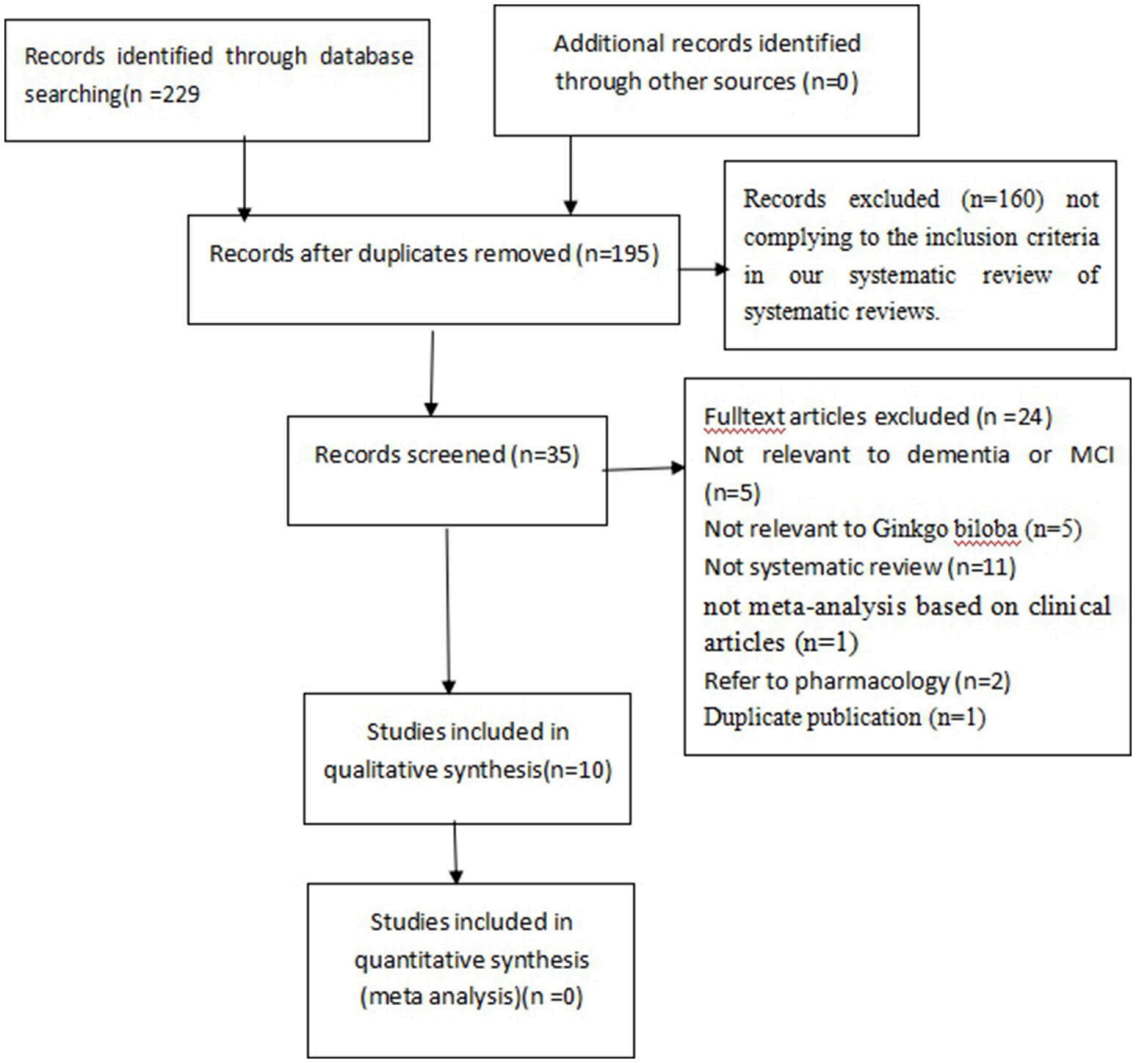
**Flow Diagram of included systematic reviews**.

The search strategy yielded 229 potentially relevant hits. After removal of duplicates, 195 records remained. Through screening titles and abstracts, we excluded 160 papers. In the remaining 35 papers, 25 papers were excluded with at least one of following reasons: (1) not relevant to dementia or MCI (*n* = 5); (2) not relevant to GBEs (*n* = 5); (3) not a systematic review (*n* = 11); (4) not a meta-analysis based on clinical articles (*n* = 1); (5) animal studies (*n* = 2); (6) duplicated publication (*n* = 1). Ultimately, 10 eligible studies were selected (Figure 1).

### Study characteristics

The 10 systematic reviews included were published between 2010 and 2015. Among them, 9 systematic reviews were published in English and 1 systematic review in Chinese (Jiang et al., 2013). 7 first authors of the 10 systematic reviews were from China (Wang et al., 2010; Yang et al., 2011, 2014, 2016; Hu et al., 2013; Jiang et al., 2013; Tan et al., 2015), 1 from Canada (Gauthier and Schlaefke, 2014), and 2 from Germany (Janssen et al., 2010; Weinmann et al., 2010). The 10 systematic reviews included reviews about Alzheimer's disease (*n* = 3), about vascular dementia (*n* = 1), about both Alzheimer's disease and vascular dementia (*n* = 2), about Alzheimer's disease, vascular dementia and mixed dementia (*n* = 3), and a review about MCI (*n* = 1) (Table 1). The inclusion criteria, the quality of randomized controlled trials, exclusion criteria, and the quality assessment in studies are presented in Table 2. Four systematic reviews (Janssen et al., 2010; Hu et al., 2013; Gauthier and Schlaefke, 2014; Yang et al., 2014) assessed methodological quality according to the Jadad score. Five systematic reviews (Wang et al., 2010; Yang et al., 2011, 2016; Jiang et al., 2013; Tan et al., 2015) used the Cochrane Collaboration guidelines to evaluate methodological quality. The remaining systematic review (Weinmann et al., 2010) assessed methodological quality according to the modified Cochrane Collaboration guidelines. The randomized controlled trials (RCTs) compared GBEs with either placebo or Western conventional medicine (WCM) in patients with MCI, VD, AD, and/or mixed dementia. The quality of randomized controlled trials in 7 studies (Wang et al., 2010; Weinmann et al., 2010; Jiang et al., 2013; Gauthier and Schlaefke, 2014; Yang et al., 2014, 2016; Tan et al., 2015) are good, while moderate in 2 studies (Janssen et al., 2010; Yang et al., 2011) and poor in 1 study (Hu et al., 2013). Seven systematic reviews (Wang et al., 2010; Weinmann et al., 2010; Yang et al., 2011, 2016; Jiang et al., 2013; Gauthier and Schlaefke, 2014; Tan et al., 2015) reported inclusion criteria and exclusion criteria, while three (Janssen et al., 2010; Hu et al., 2013; Yang et al., 2014) reported inclusion criteria only. Key data of included systematic reviews are summarized in Tables 1, 2.

**Table 1:** **Study characteristics of included systematic reviews**.

**First author**	**Condition**	**No. of primary studies**	**Quality of primary studies**	**Overall conclusion+**	**Meta-analysis**	**The nature of the extracts**	Quality of review (OQAQ)^*^	**Result +,-/±**
Yang et al., 2014	Alzheimer's disease (AD)	8	All good (8 low risk of bias)	…have significant effect on…	The total effective rate [8 Randomized Controlled Trials (RCTs)]: GBEs vs. placebo: (1) cognition outcomes: risk ratio (RR): −1.62, 95% confidence interval (CI) [−2.69, −0.52]; (2) Activities of daily living (ADL) in AD patients: RR: −1.55, 95% CI [−2.55, −0.55]; (3) Incidence of AD: RR: 1.05, 95% CI [0.91, 1.21].	Extract *Ginkgo biloba* number 761 (EGb 761), mixed GBEs	6	+
Weinmann et al., 2010	AD, vascular or mixed dementia	9	Mostly good (11 low risk of bias, 1 high risk of bias)	…have clinically meaningful effect on…	The total effective rate (12RCTs): cognition outcomes: Standard Mean Difference (SMD) = −0.58, 95% CI [−1.14, −0.01]; SMD = −0.63, 95% CI [−1.16, −0.10] for the Alzheimer subgroup; ADL outcomes: SMD = −0.32, 95% CI [−0.66, 0.03]; SMD = −0.44, 95% CI [−0.77, −0.12] for the Alzheimer subgroup.	Mixed GBEs	5	+
Tan et al., 2015	AD, vascular or mixed dementia	12	Mostly good (11 low risk of bias, 1 high risk of bias)	…positively have significant effect on…	The total effective rate (11RCTs): Cognitive function : EGb 761 for the 240 mg/day [Weighted Mean Difference (WMD) −3.19, 95% CI −3.56 to −2.83, *p* < 0.00001, 6 studies] or 160 mg/day (WMD −3.96, 95% CI −5.13 to −2.80, *p* < 0.00001, 2 studies), and all doses pooled (WMD −2.86, 95% CI −3.18 to −2.54, *p* < 0.00001, 8 studies). *I*^2^ = 96%, *I*^2^was reduced to 0% by removing (Napryeyenko et al., 2007) from the analysis. Behavioral symptoms**:** 240 mg/day EGb 761 and placebo (WMD −2.51, 95% CI −3.29 to −1.73, *p* < 0.00001, 3 studies) experiencing an adverse event: no significant differences between EGb 761 and placebo in the proportion of participants for whole group and subgroup analysis; RR:0.92, 95% CI [0.75,1.13]; experiencing a serious adverse event: (297/443 compared with 340/457) (OR 0.70,95% CI 0.52 to 0.93, *p* = 0.02, 3 studies) in AD subgroup. Safety and tolerability: There were no significant differences between EGb 761 and placebo in the proportion of participants experiencing any adverse events or serious adverse events for whole group and subgroup analysis. Among them, there was a significant difference, in favor of 240 mg/day EGb 761 compared with placebo for participants experiencing the adverse events in AD subgroup (297/443 compared with 340/457) (OR 0.70, 95% CI 0.52–0.93, *p* = 0.02, 3 studies).	EGb 761	5	+
Gauthier and Schlaefke, 2014	AD, vascular or mixed dementia	7	Mostly good (8 low risk of bias, 1 high risk of bias)	…positively have clinically meaningful effect on…	The total effective rate (8RCTs): clinical global impression: SMD −0.52; 95% CI [−0.92, −0.12], *I*^2^ = 96%; after removing the trial with the largest drug–placebo: SMD −0.33; 95% CI [−0.55, −0.11]; For adverse events: there is no suggestion of an increased risk under active treatment: RR 0.96, 95% CI [0.90, 1.01]	EGb 761	6	+
Yang et al., 2011	Mild to moderate AD	5	Partly good (5 low risk of bias)	…have significant effect on…	The total effective rate (5RCTs): When Minimum Mental State Examination (MMSE) was <24, there was a significant difference between the two groups by *U*-test (χ^2^ = 0.16, *P* = 0.69, WMD = 0.55, 95% CI = −0.77, 1.88); the Syndrom-Kurztest score (χ^2^ = 2.72, *P* <0.01, WMD = −2.32, 95% CI = −3.12, −1.52); the ADAS-cog (Alzheimer's disease assessment scale) score (χ^2^ = 2, *P* = 0.34, WMD = −4.32, 95% CI = −4.30, 1.47).	EGb 761	5	+
Wang et al., 2010	Dementia (by prospective criteria)	6	All good (6 low risk of bias)	…have clinical effect on…	The total effective rate (6RCTs): All trials but Schneider 2005 showed significant differences in favor of GBEs on ADAS-cog or Syndrom Kurz Test (SKT). Mean change differences ranged from −2.25 points (95% CI − 2.94 to − 1.55) in the Mazza 2006 trial to 0.10 (95% CI– 0.09–0.28) in the Schneider 2005 study.	Tebonin, EGb 761	5	+
Jiang et al., 2013	AD, Vascular Dementia (VD), ormixed dementia	9	Partly good (8 low risk of bias, 1 high risk of bias)	…have clinically meaningful effect on…	The total effective rate (9 RCTs): according to mean age of group members: SMD: −0.28, 95% CI [−0.51, −0.05]; according to mean daily dose of GBEs: SMD: −0.22, 95% CI [−0.43, −0.02]; Side effects: three adverse events were reported significantly less frequently in the GBEs group than in the control group: headaches (RR = 0.74, 95% CI = 0.60–0.92, *p* < 0.01, total *n* = 2060); dizziness (RR = 0.54, 95% CI = 0.30–0.97, *p* = 0.04, total *n* = 2060), and tinnitus (RR = 0.39, 95% CI = 0.24–0.65, *p* < 0.01, total *n* = 1323) while there were no statistically significant differences between the GBEs group and the control group in the occurrence of respiratory tract infections (RR = 1.09, 95% CI = 0.78–1.52, *p* = 0.62, from 4 RCT, total *n* = 1733), diarrhea (RR = 0.93, 95% CI = 0.56–1.54, *p* = 0.77, from 2 RCT, total *n* = 810), or increased blood pressure (RR = 0.73, 95% CI = 0.47–1.15, *p* = 0.17, from 3 RCT, total *n* = 1220).	Mixed GBEs	6	+
Janssen et al., 2010	AD	6	All good (6 low risk of bias)	…have significant clinical effect on…	The total effective rate (6RCTs): Ginkgo vs. Placebo: (1) ADL: Dose 120 mg: *p* < 0.001, *I*^2^ = 85.7%, Dose 240 mg: *p* < 0.001, *I*^2^ = 88.5%; (2) cognition: Dose 120 mg: *p* = 0.027, *I*^2^ = 72.3%, Dose 240 mg: *p* < 0.001, *I*^2^ = 96.7%.	Mixed GBEs	4	+
Hu et al., 2013	VD	12	Mostly poor(12 low risk of bias)	…has no effect on…	The total effective rate (2RCTs): MMSE scores: RR: 1.91, 95% CI [1.1, 2.02]; ADL scores: RR: 2.90, 95% CI [4.44, 1.35]; FAQ Score: RR: 1.93, 95% CI [3.82, 0.04].	Mixed GBEs	3	-
Yang et al., 2016	AD and MCI (mild cognitive impairment)	21	All good (21 low risk of bias)	…have significant effect on…	The total effective rate (21RCTs): Cognitive Function: Compared with conventional medicine alone, one meta-analysis favored GBEs plus conventional medicine for the treatment of AD as measured by MMSE at 24 weeks (Mean Difference(MD) 2.39, 95% CI 1.28–3.50, *P* < 0.0001, 126 participants, *I*^2^ = 0%) and two individual trials favored as measured by MMSE at 2 weeks and 16 weeks; two trials favored GBEs plus conventional medicine when measured by Hasegawa Dementia Scale at 12 weeks and 24 weeks; one trial favored GBEs plus conventional medicine as measured by SKT at 22 weeks. Compared with conventional medicine alone, one trial of GBEs for the treatment of MCI reported the original scores of cognitive function in last observation favored GBEs as measured by MMSE as 12 weeks. ADL: Compared with placebo, one trial of GBEs for AD reporting the change in scores from baseline found GBEs was superior as measured by the Geriatric Evaluation by Relative's Rating Instrument at 52 weeks. Compared with conventional medicine, one trial of GBEs for AD reporting the original scores in last observation found no significant difference between groups, as measured by the Gottfries-Brane-Steen Scale Sub-score at 22 weeks. Compared with conventional medicine alone, four individual trials and one meta-analysis reported the last observation scores of ADL: the meta-analysis found significant differences between GBEs plus conventional medicine and conventional medicine alone (MD −3.72, 95% CI −5.68 to −1.76, *P* = 0.0002, 126 participants, *I*^2^ = 51%), when measured by ADL at 24 weeks, while three individual trials found no significant difference between groups as measured by ADL at 2 weeks and at 12 weeks, and measured by GBS Sub-score of ADL at 22 weeks. Only one trial comparing GBEs with conventional medicine for the treatment of MCI reported this outcome, and found no significant difference between groups. Adverse Events: A meta-analysis of number of patients with adverse events (OR 0.82, 95% CI 0.62–1.06, *P* = 0.13, *I*^2^ = 0%) and ever adverse events (OR 0.82, 95% CI 0.39–1.74, *P* = 0.60, *I*^2^ = 0%) in trials comparing GBEs with placebo showed there was no significant difference between groups.	EGb 761, GBEs injection, Mixed GBEs	6	+

*AD, Alzheimer's disease; ADAS-cog, Alzheimer 's disease assessment scale; ADL, Activities of daily living; CI, confidence interval; GBEs, Ginkgo biloba extracts; MCI, Mild Cognitive Impairment; MD, Mean Difference; MMSE, Minimum Mental State Examination; RR, risk ratio; SKT, Syndrom Kurz Test; SMD, Standard Mean Difference; VD, Vascular Dementia; WMD, Weighted Mean Difference*.

(OQAQ)* refers to the overall score of OQAQ which is from 1 to 7.OQAQ ≤ 3, extensive or major flaws; OQAQ ≥ 5, minor or minimal flaws. Conclusion +, As judged by the authors of the respective SRs. Result+, +, overall positive; −, fail to show effectiveness; ±, unclear. Quality of primary studies regards to the systematic review which include them.

**Table 2:** **Quality assessment and inclusion and exclusion criteria of each included systematic review**.

**References**	**Quality assessment**	**Quality of randomized controlled trials**	**Inclusion criteria**	**Exclusion criteria**
Yang et al., 2014	Jadad score	Good	All published, double-blinded, randomized, placebo-controlled trials examining efficacy of natural medicine sinpatients with AD (Alzheimer's disease) were included. Diagnostic criteria included Diagnostic and Statistical Manual of Mental Disorders (DSM), National Institute of Neurological and Communicative Disorders and Stroke-Alzheimer's Disease and Related Disorders Association (NINCDS-ADRDA).	N.R:
Weinmann et al., 2010	Modified cochrane collaboration	Good	We selected controlled clinical trials, with or without randomization, assessing the effects of treating people with a diagnosis of AD, vascular or mixed dementia according to internationally valid diagnostic criteria, with a standardized *Ginkgo biloba* extracts (GBEs).	(1) Studies witha majority of people with specific types of non-vascularand non-Alzheimer's dementia, such as Lewy-bodydementia or dementia due to Parkinson's disease; (2) Apublication language other than English, German, French, Italian or Spanish.
Tan et al., 2015	The cochrane collaboration	Good	(1) Double-blind, parallel-group, placebo-controlled, with random assignment to a standardized *Ginkgo biloba* extract EGb 761; (2) Inclusion of patients who have a diagnosis of AD, vascular dementia (VD), or mixed dementia according to internationally validdiagnostic criteria for the dementia diagnosis, including the International Classification of Diseases (ICD), the Diagnostic and Statistical Manual of MentalDisorders (DSM), the NINCDS-ADRDA, or the National Institutefor Neurological Disorders and Stroke and AssociationInternationale pour la Recherche et l'Enseignement enNeurosciences (NINDS-AIREN) criteria; inclusion of patients suffering from age-associated memoryimpairment according to the diagnostic criteria proposed by Crook et al. (1990). Inclusion of patientssuffering from Mild Cognitive Impairment (MCI) according to international consensus criteria proposed by Winblad et al. (2004); (3) Inclusion of treatments which last 22–26 weeks, contain a number of participants of more than 10 pergroup, and at least one measure reflecting the following: cognition, function, behavior or global assessmentof change.	Studies with fatal flaws in study designerdata analysis were excluded, as were trials whose datawere not readily available.
Gauthier and Schlaefke, 2014	Jadad score	Good	Trials were eligible for inclusion if they were placebo-controlled, randomized, double-blind clinical trials of atleast 20 weeks induration, assessing the effects of an oral dosage form of EGb 761 in patients with a diagnosis of AD, VaD, or mixed dementia (i.e., with features of both AD andcerebrovascular disease), if the diagnosis was established inaccordance with internationally accepted diagnostic criteria DSM, Third Revised or Fourth Edition (DSM-III-R, DSM-IV),International Classification of Diseases (ICD)-10, National Institute of Neurological and Communicative Disorders and Stroke, Alzheimer's Diseaseand Related Disorders Association (NINCDS-ADRDA), or the NINDS-AIREN, 19andif outcome measures were defined for at least two of the three typical domains of assessment in dementia, i.e., cognition, activities of daily living (ADL), and clinical global judgment.	Trials including mostly patients with other diagnoses, suchas aging-associated memory impairment or mild cognitiveimpairment, and trials using EGb 761 as add-on treatmentto cholinesterase inhibitors, were excluded.
Yang et al., 2011	The cochrane collaboration (RevMan 4.2 software)	Variable	(1) Randomized placebo-controlled clinicaltrials, blind or open; (2) Over all patients: a clinicaldiagnosis of mild to moderate AD, age ≥50 years old, male and female; met the diagnostic criteria for dementia according to the American Psychiatric Association's“DSM” Fourth revised edition, mini-mental state examination(MMSE), score ≤ 26 points, mild and moderate ADpatients; (3) Outcome assessment: outcomemeasurement indicators included before and afterassessment of MMSE, ADL scale, Hasegawa Dementia Scale, Clinical Global Impression, Wechsler memory quotient scores, as well as treatmentefficiency, the number of cases and other adverse events; (4) Patients who were followed up for at least 8 weeks;the number of cases was at least 10 cases.Thetreatment group received GBEs, and thecontrol group received placebo.	(1) Original documents were not located in thecontrol group. (2) No rigorous experimental designliterature. (3) Repeated published literature. (4) Reviewof the literature. (5) Rawdata did not provide adequatedocumentation. (6) Did not contain full text of the originaldocument.
Wang et al., 2010	The cochrane collaboration	Good	(1) The study needed to be randomized, placebo-controlled, double-blind. (2) Patients needed to be diagnosed with dementia by prospective criteria. (3) The use of standardized GBEs in any stated dose was required(24% or 25% ginkgoflavone glycosides and 6% terpenoids). GBEs could be given by any route of administration. (4) At least 1 outcome measure needed to be an objectiveassessment of cognitive function. (5) Trial durations of treatment needed to be 24 ± 2 weeks ordata of assessments at 24 ± 2 weeks were available.	Studies needed to clearly state their exclusion criteria, i.e., those studies that had not stated exclusions for depression, other neurological disease, and taking central nervous system active medications were excluded.
Jiang et al., 2013	The cochrane collaboration	Good	(1) Randomized controlled trial. (2) Subjects diagnosed with AD, VD, mixed dementia using widely accepted critia. (3) Treatment group used GBEs and control group used placebo for at least 22 weeks. (4) Outcome assessed using widely accepted measures of cognitive and social function.	Studies were excluded if: (a) they were animals studies; (b) they were reviews, conference presentations, or unpublished reports; (c) they were duplicated reports; (d) other cognitive boosting medications were used as adjunctive treatments; (e) there was no placebo control; or (f) there was no control group.
Janssen et al., 2010	Jadad score	Variable	^*^Randomized controlled design. ^*^Follow-up period 16 weeks, to be able to assess along-term effect. ^*^Investigation of patients with mild, moderately severe and severe AD.Diagnosis had to be confirmedeither by the criteria of the European Medicines Agency or by commonlyaccepted ones such as ICD-9, ICD-10, DSM-II-R, DSM-IV, or NINCDS-ADRDA. ^*^Comparison of Ginkgo with placebo or other medicinal or non-medicinal interventions. ^*^Evaluation of at least one predefined patient-relevantoutcome.(In this context, the term “patient-relevant” refers to how a patient feels, functions or survives.) The following outcomes were considered: activitiesof daily living, cognitive functioning, psychopathology, quality of life and safety aspects. ^*^Language of publication: English, Dutch, French, German, Portuguese and Spanish. ^*^Availability of a full-text document (e.g., journalarticle or clinical study report). No restrictions applied for the date of publication.	N.R:
Hu et al., 2013	Jadad score	Poor	(1) Randomized controlled trialsand/or semirandomized controlled trials, regardless of whether with blind method or not andlanguageis not restricted. (2) Satisfy withdiagnosisstandard of vascular dementia, regardless ofgender, age, country. (3) Basic treatment+GBEs orwith the basic treatment +otherinterventions(a singlemedicine)	N.R:
Yang et al., 2016	The cochrane collaboration	Good	(1) Study design. Randomized control trials with at least one group involving GBEs for the treatment of AD or MCI were eligible to this review. (2) Participants. Participants diagnosed with any one of the following criteria as AD were included, regardless of severity and disease course: (a) The Diagnostic and Statistical Manual of Mental Disorder (DSM) III, III-R or IV; (b) The International Classification of Disease (ICD) (9th or 10th edition); (c) The National Institute of Neurological and Communicative Disorder and Stroke-Alzheimer's disease and Related Disorder Association (NINCDS/ADRDA). Participants diagnosed with any one of the following criteria as MCI were also included: (a) The Diagnostic and Statistical Manual of Mental Disorder (DSM) III, III-R or IV; (b) The International Classification of Disease (ICD) version 9 or 10; (c) Petersen criteria; (d) European Consortium on AD. The key differences of diagnostic criteria between AD and MCI were as follows: (1) AD can be diagnosed only if at least two domains (including memory) demonstrating cognitive impairment, while MCI can be diagnosed if there is impairment in memory; (2) AD can be diagnosed only if cognitive impairment is so severe that the ability to perform ADLs is interfered, while for MCI, there is just a slight cognitive decline and functional disturbance. (3) Interventions. Any forms of GBEs, were included. (4) Control. The control included no treatment, placebo, and conventional medications such as memantine, donepezil, galantamine and rivastigmine. Co-intervention if applied in all arms was also included. (5) Outcome measures. The primary outcomes included cognitive function and quality of life. The secondary outcomes included safety, ADL, and global clinical assessment.	(1) Study design. We excluded quasi-randomized trials. (2) Participants. We excluded trials involving participants with other types of dementia.

N.R, not reported; AD, Alzheimer's diseas; ADL, Activities of daily living; DSM, Diagnostic and Statistical Manual of Mental Disorders; EGb 761, Extract Ginkgo biloba number 761; GBEs, Ginkgo biloba extracts; ICD, International Classification of Disease; MCI, Mild Cognitive Impairment; NINCDS–ADRDA, National Institute of Neurological and Communicative Disorders and Stroke-Alzheimer's Disease and Related Disorders Association; NINDS-AIREN, National Institutefor Neurological Disorders and Stroke and AssociationInternationale pour la Recherche et l'Enseignement enNeurosciences; RCT, Randomized Controlled Trials; VD, Vascular dementia.

### Assessing the quality of systematic reviews

According to OQAQ scores, the quality of these systematic reviews varied. Nine studies included (Janssen et al., 2010; Wang et al., 2010; Weinmann et al., 2010; Yang et al., 2011, 2014, 2016; Jiang et al., 2013; Gauthier and Schlaefke, 2014; Tan et al., 2015; Table 3) were considered to have only minor or minimal flaws, i.e., scored with at least 5 points on the OQAQ. One systematic review was seriously flawed and scored 3 points only (Hu et al., 2013; Table 3). Although the review by Yang et al. (2016) received an OQAQ score of 6, this study is not useful because in the analysis it seems that no careful distinction between MCI and dementia was performed and because some important trials reporting on AD subgroups are missing (e.g., Kanowski and Hoerr, 2003; Napryeyenko et al., 2009).

**Table 3:** **The methodological quality by using the overview quality assessment questionnaire**.

	**1 Were the search methods reported?**	2 Was the search comprehensive?^*^	3 Were the inclusion criteria reported?^*^	4 Was selection bias avoided?^*^	5 Were the validity criteria reported?^*^	6 Was validity assessed appropriately?^**^	7. Were the methods used to combine studies reported?^**^	8. Were the findings combined appropriately?^**^	9. Were the conclusions supported by the reported data?^*^	**How would you rate the scientific quality of this review?**
Yang et al., 2014	Adequate	Adequate	Adequate	Adequate	Not mentioned	Not mentioned	Adequate	Adequate	Adequate	6
Weinmann et al., 2010	Adequate	Adequate	Adequate	Adequate	reported	typical for RCT assessment	Adequate	Adequate	Adequate	7.5
Tan et al., 2015	Adequate	Inadequate	Adequate	Not mentioned	Not mentioned	Not mentioned	Adequate	Adequate	Adequate	5
Gauthier and Schlaefke, 2014	Adequate	Adequate	Adequate	Adequate	reported	typical for RCT assessment	Adequate	Adequate	Adequate	7.5
Yang et al., 2011	Adequate	Inadequate	Adequate	Adequate	Not mentioned	Not mentioned	Adequate	Adequate	Adequate	5
Wang et al., 2010	Adequate	Inadequate	Adequate	Adequate	Not mentioned	Not mentioned	Adequate	Adequate	Adequate	5
Jiang et al., 2013	Adequate	Adequate	Adequate	Adequate	Not mentioned	Not mentioned	Adequate	Adequate	Adequate	6
Janssen et al., 2010	Adequate	Inadequate	Adequate	Adequate	reported	typical for RCT assessment	Not mentioned	Inadequate	Adequate	5
Hu et al., 2013	Adequate	Inadequate	Inadequate	Inadequate	Not mentioned	Not mentioned	Adequate	Adequate	Adequate	3
Yang et al., 2016	Adequate	Adequate	Adequate	Adequate	Not mentioned	Not mentioned	Adequate	Adequate	Adequate	6

* Adequate: 1; inadequate and not mentioned: 0.

** Adequate: 0.5; inadequate and not mentioned: 0.

### Effectiveness

#### Prevention

Only two systematic reviews mentioned prevention of cognitive function decline and the results were not significant. In one review (Yang et al., 2014), the preventative effect of AD were studied in two studies (DeKosky et al., 2008; Vellas et al., 2012), while in the other review (Yang et al., 2016), only one study was included (Vellas et al., 2012). However, in the study by DeKosky et al. (2008) an extremely low compliance was a serious issue; in both trials the overall conversion rate to dementia was too low to draw conclusions on the efficacy, as both studies were highly underpowered. In addition, the study by Vellas et al. (2012) was inconclusive. Not only was there a significantly lower rate of progression to AD in the prospectively specified subgroup of subjects who were on drug for at least 4 years, there was also a problem with the pre-specified statistical test which was chosen assuming proportional hazards. When this assumption turned out wrong, because hazards were found to increase during the study period, a *post-hoc* analysis was performed by Scherrer et al. (2015) which demonstrated a significantly lower rate of progression to AD in the Extract *Ginkgo biloba* number 761 (EGb 761)®-treated subjects when an appropriate statistical test was applied that accounts for the non-proportional hazards. Hence, the question whether GBE can prevent dementia or cognitive remains to open and needs to be examined in the future.

#### MCI

(1) Activities of daily living (ADL): There were no significant difference between GBEs plus WCM and WCM alone (Yang et al., 2016). (2) Cognition function: Both GBEs alone and GBEs plus WCM were more effective than WCM (Yang et al., 2016).

#### Dementia

(1) Global clinical assessment: Two systematic reviews (Wang et al., 2010; Gauthier and Schlaefke, 2014) indicated that EGb 761 was better than placebo. Gauthier and Schlaefke (2014) further demonstrated that EGb 761 was dose-dependently effective, with and high daily dose (240 mg) of EGb 761 with better efficacy compared to low dose (120 mg). (2) ADL: One systematic review (Tan et al., 2015) indicated that GBEs could improve ADL in dementia patients, but another one (Weinmann et al., 2010) did not show such effect, because that analysis could not include several of the more recent and successful trials. Jiang et al. (2013) specifically demonstrated that EGb 761 could improve ADL in relatively young (age <75 years) patients but not in those older than 75. However, only two trials were included in the age group above 75 years. When 4 trials were included in the analysis of old patients, a clear beneficial effect of EGb 761 could be demonstrated by Kasper (2015). Moreover, Jiang et al. (2013) inappropriately included the trials by van Dongen et al. (2003) and McCarney et al. (2008). van Dongen et al. (2003) included a mixed population of subjects with aging-associated memory impairment (AAMI) and dementia. Most patients had AAMI, i.e., they had–by definition–no impairment of ADL and therefore they could hardly improve significantly in ADL. In the study by McCarney et al. (2008) substantial proportions of the Ginkgo and placebo groups (about one third) were on cholinesterase inhibitors. Effective treatment with cholinesterase inhibitors may decrease the chance and the extent of further improvement by GBE. Moreover, 25% of the patients in the Ginkgo group, but only 10% of the patients in the placebo group had been treated with Ginkgo before enrollment. Those treated with GBE before are certainly less likely to show marked further improvement during the study. Furthermore, the study was seriously underpowered due to recruitment problems and due to the split of both groups to sub-groups with minimal and extensive follow-up, variances were particularly large, which further decreased statistical power. The inappropriate choice of trials for inclusion and the exclusion was seriously flawed and trials that enrolled different patients are essential for a review to mislead to the correct conclusion with regard to efficacy in a specific disorder. Two systematic reviews (Jiang et al., 2013; Gauthier and Schlaefke, 2014) demonstrated that the effects of high daily dose (240 mg) on ADL were more significant than that of placebo. Although one systematic review (Gauthier and Schlaefke, 2014) indicated that relatively low daily dose (160 or 120 mg) of GBEs could improve ADL of dementia patients, another one (Jiang et al., 2013) had opposite results. (3) Cognition function: Two systematic reviews (Weinmann et al., 2010; Tan et al., 2015) indicated that GBEs could improve cognition function in dementia patients. Furthermore, both two systematic reviews (Jiang et al., 2013; Gauthier and Schlaefke, 2014) indicated that the effects on cognition function were dose-dependent and relatively high daily dose (240 mg) of GBEs could improve cognition function in dementia patients. Jiang et al. (2013) specifically indicated that EGb 761 could improve cognition function of relatively young (age <75 years) people but not in those older than 75. However, only two trials were included in the age group above 75 years. When four trials were included in the analysis of old patients, a clear beneficial effect of EGb 761 could be demonstrated by Kasper (2015). (4) Neuropsychiatric and behavioral symptoms (NPS): GBEs improve neuropsychiatric symptoms, including depression (Weinmann et al., 2010; Jiang et al., 2013; Gauthier and Schlaefke, 2014; Tan et al., 2015), there was a significant difference in favor of 240 mg/day EGb761 (*p* < 0.00001) when assessing efficacy regarding improvement of neuropsychiatric symptoms (Tan et al., 2015).

#### AD

(1) Global clinical assessment: Two systematic reviews (Gauthier and Schlaefke, 2014; Yang et al., 2016) indicated that the effectiveness of EGb 761 was better than that of placebo. Yang et al. (2016) indicated that there was no significant difference comparing EGb 761 with WCM; however, this study is not useful because of its shortcomings explained above. (2) ADL: Four systematic reviews (Janssen et al., 2010; Weinmann et al., 2010; Yang et al., 2014; Tan et al., 2015) indicated that GBEs were effective for improving ADL. The effects on ADL were dose-dependent and relatively high daily dose (240 mg) of GBEs were more effective than placebo (Janssen et al., 2010). Yang et al. (2016) indicated that the effectiveness of GBEs was better than that of placebo but not WCM. However, due to the serious concerns regarding the review by Yang et al. (2016) mentioned above, such conclusions should be taken with a grain of salt. An additional mistake in the review by Yang et al. (2016) is that in the study by Maurer et al. (1997), there was no significant difference in the Syndrom Kurz Test (SKT) favoring EGb 761®. Actually, there was a significant difference in the SKT and there was a non-significant difference in the Alzheimer's disease assessment scale-cognitive subscale (ADAS-cog), both in favor of EGb 761®. The size of the change in the ADAS-cog was as expected from the size of the change in the SKT (both correlate very well), but due to the variance this difference was not significant for the ADAS-cog in the very small number of patients. In the study by Mazza et al. (2006), there also was a significant difference in favor of active treatment in the SKT, but not in the MMSE. The MMSE is a screening test for cognitive impairment and dementia and has never been validated as an outcome measure for treatment trials in dementia. Actually, in the study by Mazza et al. (2006) the difference in MMSE changes between donepezil and placebo was not significant either. These issues demonstrate again that assessing the quality of a review always involves a careful evaluation of the quality of the single trials and their correct representation in the review. (3) Cognition function: Two systematic reviews (Weinmann et al., 2010; Tan et al., 2015) indicated that GBEs improve cognition function in AD patients. (4) NPS: In an earlier analysis that could not yet take into account the most recent clinical trials, no significant effect on neuropsychiatric and behavioral symptoms could be demonstrated (Weinmann et al., 2010). However, in a more recent review, a significant effect of EGb 761 on neuropsychiatric symptoms could be demonstrated (Tan et al., 2015). (5) Quality of life: Yang et al. (2016) indicated that GBEs for AD was superior to placebo as measured by the quality of life questionnaire for person with dementia (DEMQOL)-proxy quality of life scale. Janssen et al. (2010) indicated that two trials available in that domain at the time that review was generated showed contradictory results. VD (Hu et al., 2013): (1) ADL: GBEs were as effective for improving ADL of VD patients compared with WCMs such as donepezil, gamma aminobutyric acid (GABA)-ergic drugs or other therapies. (2) Cognition function: GBEs could improve cognition function in VD patients according to Minimum Mental State Examination (MMSE) scores.

### Adverse events

Eight systematic reviews (Janssen et al., 2010; Weinmann et al., 2010; Yang et al., 2011, 2016; Hu et al., 2013; Jiang et al., 2013; Gauthier and Schlaefke, 2014; Tan et al., 2015) examined adverse effects, while the other two (Wang et al., 2010; Yang et al., 2014) did not. Yang et al. (2011) reported that one patient had hypersensitivity. Weinmann et al. (2010) concluded that there was no difference between the GBEs group regarding adverse events and the placebo group. Janssen et al. (2010) found that, although the number of patients that discontinued the trial was higher compared to the placebo group, no evidence for a harmful effect of GBEs was observed. Interestingly, GBEs potentially reduce adverse events. Jiang et al. (2013) reported that headache (RR = 0.74, 95% CI = 0.60–0.92, *p* < 0.01, total *n* = 2060); dizziness (RR = 0.54, 95% CI = 0.30–0.97, *p* = 0.04, total *n* = 2060), and tinnitus (RR = 0.39, 95% CI = 0.24–0.65, *p* < 0.01, total *n* = 1323) were significantly less frequent in the GBEs group than in the control group. There were no significant differences in the occurrence of respiratory tract infections (RR = 1.09, 95% CI = 0.78–1.52, *p* = 0.62, from 4 RCT, total *n* = 1733), diarrhea (RR = 0.93, 95% CI = 0.56–1.54, *p* = 0.77, from 2 RCT, total *n* = 810), or blood pressure elevation (RR = 0.73, 95% CI = 0.47–1.15, *p* = 0.17, from 3 RCT, total *n* = 1220) between the GBEs group and the control group. Tan et al. (2015) indicated that occurrence of dizziness, tinnitus, headache and angina pectoris in the GBEs group was lower compared to the placebo group. Gauthier and Schlaefke (2014) indicated that GBEs have no increased risk for headache, dizziness, hypertension, tinnitus, angina pectoris or respiratory tract infection. Yang et al. (2016) indicated that there was no significant difference in nausea, dizziness, headache, blood pressure elevation, respiratory tract infection, dyspepsia, epigastric discomfort, weight loss, agitation, constipation, diarrhea, dry mouth, and bradycardia between the GBEs and the placebo group. Hu et al. (2013) reported that there was no difference in dizziness, headache, rash, insomnia, and diarrhea between the GBE and the placebo group.

## Discussion

### Summary of evidence

Ten systematic reviews were included in the present study. Eight out of the ten systematic reviews had high quality according to OQAQ. To our knowledge, this is the first systematic review for systematic reviews on efficacy and safety of GBEs for MCI and dementia. Medication with GBEs showed improvement in cognition and daily activities, and the effect was dose-dependent. Significant efficacy was only demonstrated when they were used in high daily dose (240 mg). Compared with placebo, adverse events were fewer in patients treated with GBE compared with placebo regarding the symptoms of dizziness/vertigo, tinnitus, headache, and angina pectoris. GBEs improve cognition functions but had no effect on ADL in MCI patients. However, there may be subtle impairment in instrumental ADLs in MCI patients, and then the scales used in earlier trials were certainly not sensitive enough to detect the presence or treatment-related changes in such subtle instrumental ADLs. There is no use measuring ADLs in patients with MCI or AAMI who have subtle or no ADL impairment, and then saying there was no improvement. GBEs improve NPS both in dementia patients and in the subgroup of AD patients. GBEs improve quality of life but couldn't change the progression of AD patients. However, the effects of GBEs on the progression of AD pathology have never been studied. Hence, it is not known if GBEs have such a disease-modifying effect, but what has not been studied cannot just be denied.

### Limitations

Some weaknesses exist in this study. (1) We also included reviews that did not clearly distinguish between Ginkgo extracts generated using different extraction methods. Extracts derived from the same plant can display substantial differences in composition, efficacy and safety. For instance, differential effects of EGb 761 have been demonstrated by Itil et al. (1996) when compared with other Ginkgo extracts. Efficacy of Ginkgo extracts was only demonstrated for the special extract EGb 761. (2) Even though most of these systematic reviews have high quality, the methodology of some primary trials may not be totally scientific which may result in some contradictory results. (3) By analyzing systematic reviews rather than clinical trials, important details of the primary studies may have been lost. (4) The weakness rested with primary studies. The objective of a systematic review is to provide a complete, exhaustive summary of current literature according to presetting a research question. The most important step is a thorough search of the relevant literature when conducting a systematic review. Thus, missing the important literature would undermine. In the present study, only a few systematic reviews included the large, recent trials by Ihl et al. (2011) and Herrschaft et al. (2012). Consequently, some reviews included here lack vital evidence and yielding inaccurate results.

### Implications for future research and practice

Our study provides a broad summary on all systematic reviews of GBEs for prevention, MCI and dementia which was essential for determining the efficacy of multipletherapeutic interventions, providing an important source for clinical and health policy decision making, as well as for clinical guidelines. GBEs are applied to the entire process of dementia, from prevention, MCI to dementia severe cases. In addition, GBEs seemed to be generally safe for clinical application.

Up to now, no drug has been demonstrated to prevent cognitive function declining and progression to AD in healthy people. However, in a 20-year long follow-up population-based study, Amieva et al. (2013) indicated that EGb 761 specifically prevented cognitive decline in a non-demented elderly population when compared with non-users. Thus, the long effects of GBEs on preventing cognitive function decline need to be further confirmed.

One systematic review focused on the MCI, indicating that there is mixed evidence to support efficacy of GBEs for MCI. A well designed RCT demonstrated that *Ginkgo biloba* extract EGb 761 improved cognitive functioning and aspects of quality of life in very mild MCI patients when compared with placebo control (Grass-Kapanke et al., 2011), and another one in MCI patients with neuropsychiatric symptoms (Gavrilova et al., 2014). However, no primary systematic review included this important RCT. Furthermore, a correct diagnosis is a crucial issue in every field lacking objective/instrumental markers of the disorder/syndrome under investigation (Halbreich, 2004). Based on the Diagnostic and Statistical Manual (DSM)-5 of the American Psychiatric Association, cognitive decline is divided into major and mild neurocognitive disorders (American Psychiatric Association, 2013). The DSM-5 classification has the advantage of covering the full range of MCI and dementia on the one hand and the different etiologic types of neurocognitive disorders on the other. However, the DSM-5 is not the only classification and diagnostic system that is appropriate to diagnose and select patients for clinical trials. A recent paper by Hoerr and Zaudig (2016) shows that the patients enrolled in the dementia trials meet the DSM-5 diagnostic criteria for major neurocognitive disorders and the patients enrolled in the recent MCI trials (as well as some older trials) meet the DSM-5 criteria for mild neurocognitive disorders. Thus, at least for the special Ginkgo extract EGb 761®, there is no need to do new trials in patients defined by the DSM-5. Further rigorous RCT on GBEs should be performed according to appropriate diagnostic criteria.

There is clear evidence to support efficacy of GBEs for dementia, and the effect is dose and age-dependent. Efficacy was only demonstrated when they were used at a high daily dose (240 mg), and efficacy was only demonstrated for the extract EGb 761. We recommend that dementia patients with the above characters can choose EGb 761 as a treatment. Regarding prevention of dementia in healthy people, it should be remembered that a lack of scientific evidence does not necessarily mean that the treatment is ineffective (Kotsirilos, 2005). Whether healthy patients at risk or lower doses of GBE, or Ginkgo extracts other than EGb 761 are effective, still needs to be evaluated by rigorous clinical trials using the best available scientific standards. The patients should be divided into more subgroups according to different ages, GBEs dosages, and DSM-5 classification criteria of cognitive decline.

## Conclusion

The interests of the public and the medical profession in the use of GBEs for MCI and dementia have grown considerably in recent years. Our analysis supports the efficacy of GBEs for MCI and dementia of both the Alzheimer type and the vascular type of dementia, and of mixed dementias. In addition, GBEs are generally safe.

## Author contributions

HZ, YZ, LH, QZ, HW, GZ, and YL designed the study; HZ and YZ collected the data; HZ and YZ performed all analyses; HZ, YZ, GZ, and YL wrote the manuscript. All authors contributed to writing of this manuscript.

### Conflict of interest statement

The authors declare that the research was conducted in the absence of any commercial or financial relationships that could be construed as a potential conflict of interest.
